# New trends in single-molecule bioanalytical detection

**DOI:** 10.1007/s00216-020-02540-9

**Published:** 2020-03-17

**Authors:** Eleonora Macchia, Kyriaki Manoli, Cincia Di Franco, Gaetano Scamarcio, Luisa Torsi

**Affiliations:** 1 Center for Functional materials, The Faculty of Science and Engineering, Åbo Akademi University, 20500 Turku, Finland; 2 Dipartimento di Chimica, Università degli Studi di Bari “Aldo Moro”, 70125 Bari, Italy; 3 CNR - Istituto di Fotonica e Nanotecnologie, Sede di Bari, 70125 Bari, Italy; 4 Dipartimento di Fisica, “M. Merlin” – Università degli Studi di Bari “Aldo Moro”, 70125 Bari, Italy; 5 Centre for Colloid and Surface Science (CSGI), 70125 Bari, Italy

**Keywords:** Organic bioelectronic electrolyte-gated large transistors, Single-molecule bioanalytical technologies, Organic bioelectronics

## Abstract

Single-molecule sensing is becoming a major driver in biomarker assays as it is foreseen to enable precision medicine to enter into everyday clinical practice. However, among the single-molecule detection methods proposed so far, only a few are fully exploitable for the ultrasensitive label-free assay of biofluids. Firstly introduced single-molecule sensing platforms encompass low-background-noise fluorescent microscopy as well as plasmonic and electrical nanotransducers; these are generally able to sense at the nanomolar concentration level or higher. Label-based single-molecule technologies relying on optical transduction and microbeads that can scavenge and detect a few biomarkers in the bulk of real biofluids, reaching ultralow detection limits, have been recently commercialized. These assays, thanks to the extremely high sensitivity and convenient handling, are new trends in the field as they are paving the way to a revolution in early diagnostics. Very recently, another new trend is the label-free, organic bioelectronic electrolyte-gated large transistors that can potentially be produced by means of large-area low-cost technologies and have been proven capable to detect a protein at the physical limit in real bovine serum. This article offers a bird’s-eye view on some of the more significant single-molecule bioanalytical technologies and highlights their sensing principles and figures-of-merit such as limit of detection, need for a labelling step, and possibility to operate, also as an array, directly in real biofluids. We also discuss the new trend towards single-molecule proof-of-principle extremely sensitive technologies that can detect a protein at the zeptomolar concentration level involving label-free devices that potentially offer low-cost production and easy scalability.

## Introduction

To have the chance to collide into each other and eventually react in a reasonably short time period, two reacting species, both in solution or one in solution and one anchored on a surface, should be restrained in an adequately small volume. A volume of 1 μm^3^ (1 femtoliter, fl) is small enough, for example, for a single enzyme to interact with its substrate on the minute timescale [1]. Since a solution comprising *n* = 1 ± 1 (√*n *= Poisson error) molecules in each 1-fl subvolume has a concentration of ca. 1 × 10^−9^ mol l^−1^ (nM), one approach is to have one of the two species present at nanomolar concentration or higher. Let us consider an antibody and its cognate antigen as the two reacting species. The single antibody is bound to a surface whose size is suitable to be hosted into a 1-fl volume, while the antigen is in the solution to be assayed. They will engage in a single-molecule interaction in a reasonable time frame if the concentration of the antigen solution is nanomolar or higher. A single-molecule interaction will occur, likely with a higher probability, if many antigens are attached to the surface serving as the detecting interface. By contrast, the smaller the area of the detecting interface is, the less antibodies can be hosted and the lower the interaction rate is. So, if the detecting interface is nanometric, even smaller volumes (attoliter, al; or zeptoliter, zl) or equivalently higher concentrations should be adopted.

Samples aliquots of 100 μl of different standard solutions are generally used for clinical assays as the maximum uncertainty in measuring and transferring such a volume is 1%. Since the number of molecules in a volume* V* of a solution of molar concentration* c* is* N* = *c*·*V*·*N*_A_ (*N*_A_ = Avogadro’s number), 1 nM in* V* = 100 μl is equivalent to about 6 × 10^10^ molecules. Every 1.6 fl statistically contains one reagent, so wherever the other single reagent is, there is always a subvolume of 1.6 fl containing both reagents. Indeed, two approaches can be adopted to selectively detect a single molecule in a reasonably short time frame: (a) few recognition elements are engaged to detect biomarkers or analyte molecules present at the nanomolar level in a solution to be analyzed; (b) a large number of recognition elements, present at nanomolar concentration (or higher), are used to scavenge a single analyte in the large 100-μl volume. In supersensitive assays, only the latter approach should be used. However, to assay a 100-μl sample containing one single analyte, a platform with a limit of detection of 10 zM needs to be used, which is challenging. On the other hand, approaches entailing few recognition elements need analyte molecules at nanomolar concentration or higher. Hence, the technologies based on the latter approach are not suitable to sense a single molecule in the bulk milieu. Moreover, a number of high-throughput analytical-clinical methods can already detect biomarkers at the nanomolar level or lower. Among others there is the enzyme-linked immunosorbent assay (ELISA) platform [2].

This trends article offers a bird’s-eye view of a selection of single-molecule bioanalytical technologies and discusses some very recent new trends along with more commonly adopted approaches. The reviewed technologies encompass the fluorescent microscopy from the early days of single-molecule detections, a number of plasmonic and electrical nanotransducers to cover new trends that include extremely sensitive technologies such as the already commercialized biomarker digital-tracking microbeads label-based platforms as well as the very recent proof of concept involving label-free organic bioelectronic large sensors detecting at the physical limit. These platforms, which can all selectively detect the analyte of interest, are discussed on the basis of key figures of merit such as limit of detection, presence of a labelling step, and possibility to operate, also as an array, directly in real biofluids.

## Label-based far-field single-molecule imaging

High-quality optics enable one to restrain the focus of a visible laser beam at the diffraction limit into a volume of ca. 1 fl. Hence, a fluorogenic single-molecule or a suitably labelled one falling in this volume can be detected with low-background-noise fluorescent techniques such as epifluorescence, confocal and total internal reflection fluorescent microscopy. This is generally referred to as the label-based far-field approach to single-molecule detection. The labelling step is the operation needed to add a further element (in this case the fluorescent label) other than the biological recognition element and the molecule to be analyzed. The label is needed to make the recognition event detectable and can be also an advantage as it can improve selectivity and enable multiplexing. Indeed, in clinical practice, to gather enough information to formulate a diagnosis, a number of biomarkers are normally quantified from the same biological sample during the same measurement assay. This is called multiplexing [3] and requires a technology amenable to be developed into an array of 96 or more transducing elements, so that the standard solutions, the negative controls, and the sample can be assayed with all the replicates for each biomarker at the same time.

**Fig. 1:**
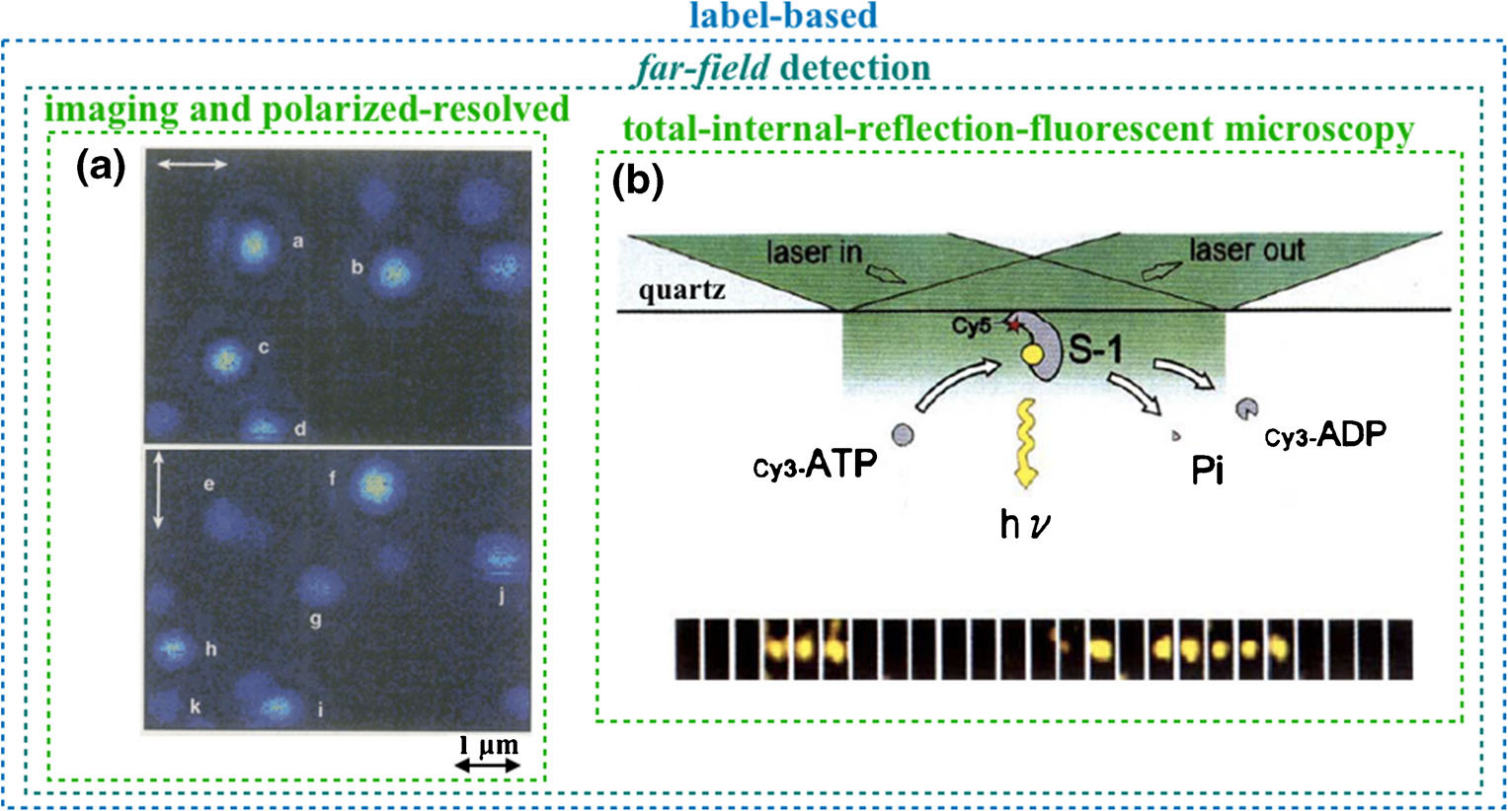
Label-free far-field single-molecule detections. **a** Sequential fluorescence images (top and bottom panels) of the same sample area showing the fluorescence image from single dye molecule inclusions (marked with letters a–i) in a PMMA film at a PMMA–air interface; images are taken with 532-nm laser excitation polarized as indicated by the white arrows. (Reprinted with permission from Ref. [4] Copyright 1996 Science). **b** The top panel shows a representation of the total internal reflection fluorescent microscopy principle for a single myosin subfragment (S-1; recognition element) that selectively binds adenosine triphosphate (ATP). ATP hydrolysis generates adenosine diphosphate (ADP) plus a phosphate (Pi) yielding energy; the bottom panel shows the measurement of an individual fluorescently labeled ATP molecule during the elicited turnover reaction. The ATP is seen only when in the proximity of the quartz interface. (Reproduced with permission from Ref. [5] Copyright 1995 Springer Nature)

In the early days of single-molecule detection, fluorescence images of single dye molecules located at a polymer–air interface were produced with an epi-illumination scanning microscope using a focused 532-nm exciting laser beam [4]. The sample comprised randomly oriented carbocyanin molecules (ca. 1 per μm^2^) in a poly(methyl methacrylate) (PMMA) film. Fluorescence images of the dyes in the focused laser spot are shown in Fig. 1a. Specifically, the images of the same area show the fluorescent single molecules acquired sequentially with differently polarized (white arrows) laser excitations. The shifts in the fluorescence spectrum could be correlated to changes in the excited state lifetime induced by the orientation of the transition dipole moment of each single molecule.

The total internal reflection fluorescent image of a fluorescently labeled single adenosine triphosphate (ATP) turnover reaction is shown in Fig. 1b. Single-headed myosin subfragments (S-1), serving as the recognition elements, are attached to quartz high-index material. ATP selectively binding to myosin is eventually hydrolyzed to adenosine diphosphate (ADP) while a phosphate (Pi) and energy are produced. To follow a single ATP turnover to ADP, the exciting laser light impinging beyond Brewster’s angle is focused on one single S-1 (Fig. 1b, top). The evanescent wave is hence localized at the interface between the quartz and the 10 nM Cy3-labelled ATP solution. The Cy3-ATP coming in and out of focus in the turnover events (Fig. 1b, bottom) can be eventually observed as the background fluorescence is minimized [5].

Important novel trends in digital optical bioassay also include wide-field epi-fluorescence excitation [6] or label-free methods to study biomolecular interactions by means of optical biosensors [7].

## Label-free near-field detection of single-molecule interactions

**Fig. 2:**
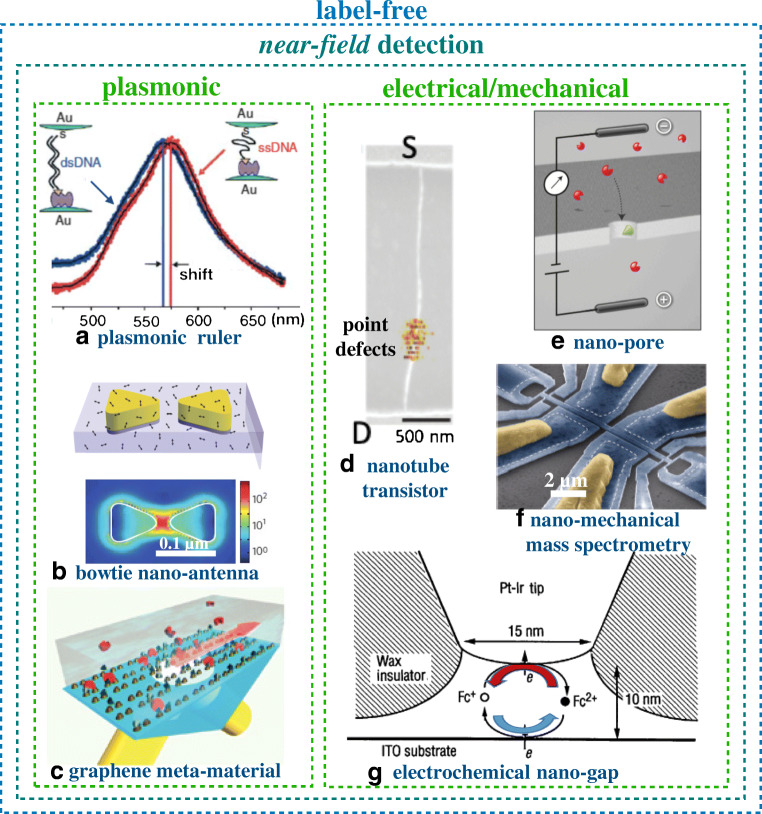
Single-molecule assays with near-field detections. **a** Spectral shift of the plasmonic signal generated by a pair of gold particles connected by a single-stranded DNA (red curve) and a double-stranded DNA (blue curve) (Reprinted with permission from Ref. [9] Copyright 2005 Springer Nature). **b** Schematic structure of a gold bow tie nanoantenna in a thin poly(methyl methacrylate) film doped with a near-infrared* N*,*N*′-bis(2,6-diisopropylphenyl)-1,6,11,16-tetra-[4-(1,1,3,3-tetramethylbutyl)phenoxy]quaterrylene-3,4:13,14-bis(dicarboximide) (TPQDI) dye (top panel) and the result of the modelling of local intensity enhancement in the bow tie structure (bottom panel) (Reprinted with permission from Ref. [10] Copyright 2009 Springer Nature). **c** Schematics of the biosensing apparatus entailing a plasmonic metamaterial. Biotin (recognition element) is represented by dark green triangles and streptavidin (analyte) by red disks. Light beams are shown in yellow while the grazing diffracted wave is represented by a red arrow (Reprinted with permission from Ref. [11] Copyright 2013 Springer Nature). **d** The single carbon nanotube transistor is shown along with the source (S) and drain (D) pads. The gating is performed through a phosphate-buffered saline solution at pH 7.4. The electrochemically generated point defects serve as anchoring sites to covalently bind single-stranded DNA probes (Reprinted with permission from Ref. [12] Copyright 2011 Springer Nature). **e** Cross-sectional sketch featuring a nanopore system through which charged single molecules translocate. Charged analyte molecules are depicted in red while the recognition element is colored in green (Reprinted with permission from Ref. [13] Copyright 2012 Springer Nature). **f** Electron micrograph of a multimode nano-electromechanical based mass detection. Yellow regions represent Al/Si gate contacts. Narrow gauges near the ends of the fingers become strained with the motion of the finger, thereby transducing mechanical motion into electrical resistance (Reprinted with permission from Ref. [14] Copyright 2012 Springer Nature). **g** Schematic illustration of the tip–substrate configuration in electrochemical nanowells engaging one electroactive species (Reprinted with permission from Ref. [15] Copyright 1995 Science)

Label-free investigations reaching single-molecule resolution have been performed, so far, by means of nanometric probes. These are referred to as near-field approaches [8] involving, for instance, tip-enhanced imaging and/or spectroscopic techniques. Here the tip apex size, comparable to the probed object, enables lateral resolutions beyond the diffraction limit. A plasmonic nanofocusing structure (antenna) that supports strong local field enhancement and confinement is used to enable the imaging of single-molecule events. The hybridization of a single DNA strand (the probe), connecting two gold nanoparticles, that is detected as a plasmonic resonance shift by transmission dark-field microscopy is featured in Fig. 2a [9]. The nanometric distance between the two nanoparticles changes when they are pulled apart as the probe is hybridized by a complementary DNA strand present at a concentration of 100 μM. In Fig. 2b shows a gold bow tie nanoantenna that produces a thousand times enhancement of single-molecule fluorescence [10]. An array of bow ties is coated with a PMMA film doped with a high concentration, ca. 3 molecules in (10 nm)^2^, of a dye (Fig. 2b, top). A high contrast image of a single dye is enabled by enhanced absorption and an increased radiative emission rate (Fig. 2b, bottom), leading to an order of magnitude higher intrinsic quantum efficiency. Metamaterials with diffractive coupling of localized plasmon resonances were devised to create complete darkness (low background) resulting in a plasmon resonance high phase sensitivity [11]. Such a metamaterial, integrating biotin recognition elements conjugated to graphene, was engaged to extrapolate the detection of an interaction with streptavidin, serving as analyte, present at a concentration of 10 pM (Fig. 2c).

The label-free near-field approach to single-molecule detection can be extended to nanometric transducing interfaces beyond plasmonic applications. A paradigmatic example is the carbon nanotube field-effect transistor (FET) detection of a single DNA strand (Fig. 2d). The nanotube bears, covalently attached to a point defect, a few single-stranded DNA probes complementary to the strand serving as analyte. The current flowing in the nanotube through the source (S) and the drain (D) contacts, gated through an electrolyte, is measured [12]. When one of the analyte strands of the 1-μM solution in contact with the nanotube hybridizes, the probe forms a complex and the conductance of the nanotube changes. In Fig. 2e a nanopore of a membrane that separates two electrolyte-filled compartments is shown. A transmembrane bias electrokinetically drives the charged analytes through the pore to generate transient blockades in the transpore current. When the pore is chemically modified with a weakly selective recognition element (nitrilotriacetic acid), stochastic sensing of a protein, at concentrations of 50–100 nM, is observed* in operando* [13]. Also of interest is the single-molecule mass spectrometry based on a nano-electromechanical system that is sensitive to the inertial mass of neutral particles that adsorb on the resonators (Fig. 2f). As each molecule in the sample adsorbs on the resonator, its mass and adsorption position are determined by continuously tracking the device vibrational modes [14]. Here the concentration of the immunoglobulin M analyte is as high as 6 μM. Electrochemistry can serve also as a transducing platform for single-molecule detection [15, 16]. A nanowell of ca. 10 nm formed between a conductive tip and a substrate is shown in Fig. 2g [15]. Here [(trimethylammonio)methyl]ferrocene (Cp2FeTMA^+^) was used as the target molecule. When the tip was held at its electrochemical potential, the Cp2FeTMA^2+^generated at the tip was rapidly reduced back to Cp2FeTMA^+^ and large step-like current fluctuations were observed. These were ascribed to the faradaic current produced by individual molecules residing in the nanowell region.

## Far- and near-field approaches are not suitable for single-molecule clinical assays

Far- and near-field approaches involve few recognition elements as their number is limited by the small volume inspected at the microscope focus (far-field) or by the transducing nanointerfaces (near-field). The lateral resolution in far-field mode is at the exciting laser diffraction limit defining a volume of 1 μm^3^ or 1 fl; near-field mode allows one to improve the resolution down to 10–100 nm, corresponding to a volume of ca. 1 zeptoliter (zl) to  1 attoliter (al). These types of methods often are also referred to also as “stochastic” sensing. Although such a dramatic reduction of the interaction volumes limits the accessible concentrations of analytes to the picomolar to millimolar range (10^8^–10^17^ molecules in 100 μl), which can be seen as a drawback, this approach is very attractive to spot rarer events. As a representative case, we can consider the nanotube transistor shown in Fig. 2d, which can detect jumps of the conductance value between the two levels associated with binding/unbinding events of a single analyte DNA strand. These experiments are carried out using 1-μM solutions, corresponding to ca. 2 × 10^15^ DNA biomolecules in 100 μl, or else ca. 20 analytes in the attoliter volume probed by the near-field detecting interface. Significantly, single-event response allows one to assess key interaction parameters, such as the rate constants and activation energies of rarer events and to compare them to those of larger ensembles. Actually, it is a fact that either far- or near-field schemes cannot give a response at the 10-zM limit of detection in a reasonable time, because the probability of placing the tiny detecting probe (e.g., the focused beam waist of the microscope objective or the transistor nanointerface) in the 1 fl–1 al subvolume in the inspected 100-μl bulk is negligibly small.

## Wide-field capturing in commercialized label-based single-molecule clinical assays

An alternative approach to detect a single molecule is to largely increase the number of recognition elements. Platforms such as Single Molecule Array (Simoa™) [17] by Quanterix [1, 18], Single Molecule Counting (SMC™) Erenna® by MilliporeMerck [19], and Ion Torrent™ by ThermoFisher [20] are indeed among the most sensitive, successfully commercialized platforms for clinical analysis. Whilst talking about sequencing methods, high-performance techniques such as Illumina MiSeq [21], Illumina NextSeq [22], and Pacific BioSciences [23] should also be mentioned. They hold the potential to revolutionize the way healthcare is provided and are paving the way towards the broad use of precision medicine in everyday clinical practice. The operation principle, referred to as wide-field capturing [8], generally involves partitioning the detecting interface into an array of a large number of microreactors (microwells), allowing most compartments to be loaded with zero or one target molecule. Alternatively, a large number of microbeads whose surface is covered (biofunctionalized) by the recognition elements are dispersed into the bulk of the biofluid to seek and bind the few biomarkers to be assayed. The beads are then loaded on an array of size-exclusion microwells accommodating zero or one bead.

**Fig. 3:**
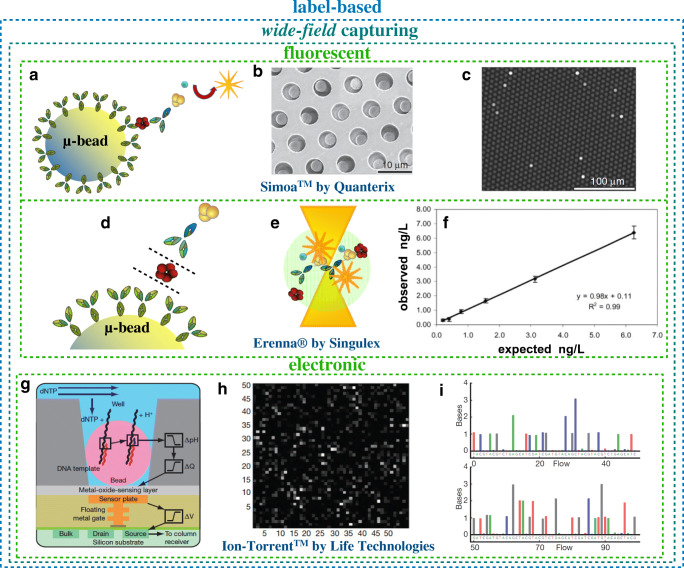
Wide-field capturing technologies. **a** Microbead biofunctionalized with specific capture antibodies according to the three-step ELISA detection strategy. **b** Scanning electron micrograph of a portion of a femtoliter-volume size-exclusion microwell Simoa™ array after bead loading. **c** Fluorescence image of the array showing the signals generated from single beads that are hosted in different wells (panels** a**,** b**, and** c** are reprinted with permission from Ref. [18] Copyright 2010 Springer Nature). **d** A magnified view of the biofunctionalized magnetic microbead. **e** A reproduction of fluorescently labelled antibodies to be counted while they pass through the confocal microscope focus. **f** Cardiac troponin I concentration is measured using standard solutions (observed concentration) of known nominal concentration (expected concentration). Concentrations are given in nanograms per liter; given that the molar mass of cardiac troponin is 22.5 kDa, this is equivalent to 40 fM. (Reprinted with permission from Ref. [19] HighWire Press; American Association for Clinical Chemistry, copyright 2007). **g** Pictorial reproduction of an Ion Torrent pixel, see text for details. **h** Picture of 2500 wells with bright intensity indicating the number of bases incorporated per well at a given nucleotide flow. **i** Chart indicating the first 100 nucleotide flows for one well with each bar height corresponding to the number of nucleotides incorporated; the color code accounts for the different nitrogenous basis: adenine (A, green), guanine (G, gray), thymine (T, red) and cytosine (C, blue) (panels** g**,** h**, and** i **are reprinted with permission from Ref. [20] Copyright 2011 Springer Nature)

In a Simoa™ protein assay [1], as many as 2 × 10^5^ magnetic microbeads biofunctionalized with 2.5 × 10^5^ specific capturing antibodies (Fig. 3a) are engaged. The concentration of the specific capturing antibodies is ca. 1 nM, so about 70% of the few biomarkers can be captured in a time frame of minutes. Afterwards, similar to ELISA, a second antibody labelled with an enzyme is allowed to bind to the biomarker. The beads are then loaded on an array of size-exclusion microwells (Fig. 3b) accommodating zero or one bead. The microwells are sealed in the presence of the fluorogenic substrate serving as label, activated only in the presence of the enzyme and, hence, of the biomarker. The detection of the loaded beads is carried out by counting the fluorescent wells (Fig. 3c) with a white light scattering captured by a standard CCD camera. Limits of detection are reported to be in the 10-aM range (thousands of proteins in 100 μl) [18] or lower [24].

The Erenna® immunoassay system [19] performs one-by-one counting of protein biomarkers. After the binding of the biomarkers (step 1), the biofunctionalized magnetic microbeads undergo a fluorescent labelling (step 2), as in Simoa™. The magnetic beads are gathered (step 3) and the fluorescent complexes are detached from their surface (step 4, Fig. 3d). By means of a capillary fluidics, the fluorescent complexes are conveyed into a 5-μm chamber located at the objective focus of a confocal microscope (step 5, Fig. 3e). Propriety software counts the biomarker proteins by correlating the detected photons emitted and the number of fluorescent events. In Fig. 3f the measured (observed) concentration of standard solutions of cardiac troponin I is plotted vs. their known nominal (expected) concentrations. The quality of the dose curve is very good (slope = 0.98 and* R*^2^ = 0.99%) with limits of detection of 10–100 pg l^−1^, equivalent to 10–100 aM [19]. Both Simoa™ and Erenna® are label-based methods and can assay real biosamples. While Simoa is an array amenable to multiplexing, with Erenna® different biomarkers must be assayed sequentially.

To assay genomic biomarkers at the physical limit by sequencing them, among others, the Ion Torrent™ next-generation sequencing [20] can be used as well as the single-molecule, real-time sequencing developed by Pacific BioSciences that offers longer read lengths [23]. In the former approach, biomarker strands are adsorbed and amplified on the surface of microbeads that are loaded into a chip of size-exclusion wells comprising a pH-sensitive MOSFET at the bottom. Figure 3g shows a schematic of a pixel of the chip and one biomarker (black strand) on the bead is featured along with the template (red strand) that starts the sequencing process through the polymerase DNA (or RNA) synthesis. The chip is sequentially flooded with one of the four nucleotides (deoxyribose nucleotide triphosphate, dNTP) and, once the nucleotide reorganizes its complementary strand, it is incorporated (blue strand) in the growing red strand. This involves the hydrolysis of the incoming nucleotide that releases protons (H^+^) so the pH of the solution changes (ΔpH). Eventually, a change in the surface potential of the metal oxide sensing layer and a shift in MOSFET threshold voltage (Δ*V*) occur. Figure 3h shows the number of bases incorporated per well for a given nucleotide flow. Figure 3i shows the first hundred nucleotide flows for one well, with each bar indicating the number of nucleotides incorporated. Being a sequencing technique, Ion Torrent can enable not only the detection of biomarkers but also the identification of the whole genomic material in a cell. This is enabling single-cell analyses that will allow one to uncover rare cell populations and unveil tumor features that cannot be discerned from conventional bulk studies. While this wide-field capturing technique can assay biofluids for clinical analysis at the single-molecule level, it is label-based because it relies on the action of the polymerase enzyme. This can be considered as a drawback. On the other hand, the repeatability and precision figures of merit for these already commercialized technologies are extremely already high, while their suppliers have very active research activity going on at their premises.

## Wide-field, label-free single-molecule sensing with electrolyte-gated field-effect transistors

Wide-field sensing [8] involves the assay of an analyte at the attomolar or even zeptomolar limit of detection with a wide interface hosting a large number of recognition elements (10^11^–10^12^ cm^−2^). A sensing technology capable of selectively detecting the change of an intrinsic property upon formation of a recognition element–analyte complex is generally referred to as label-free. For instance, in the case of an electronic detecting interface, the electrostatic or dielectric property changes have been successfully addressed. While these are strong motivations underpinning the study of organic bioelectronic sensors for ultrasensitive detection, the challenge is to perform the detection with a sufficiently high signal-to-noise ratio. Among the most interesting and high-performance bioelectronic sensors one can find the field-effect transistor (FET) devices, gated via an ionically conducting and electronically insulating electrolyte. These are referred to as electrolyte-gated FETs (EG-FETs) [25] and are generating a lot of interest as they are foreseen to be produced by scalable large-area low-cost approaches [26, 27]. These sensors [28–30] are endowed with selectivity via the biofunctionalization of the semiconductors (SCs) [31] or the gate surface [28, 29, 32]. EG-FETs based on electronic channel materials such as graphene [33], poly(3,4-ethylenedioxythiophene) polystyrene sulfonate (PEDOT-PSS) [34], and poly(3-hexylthiophene-2,5-diyl) (P3HT) [35–39] have been successfully engaged, lately, as wide-field organic bioelectronic sensors exhibiting limits of detection at zeptomolar to attomolar level also in real biofluids.

**Fig. 4:**
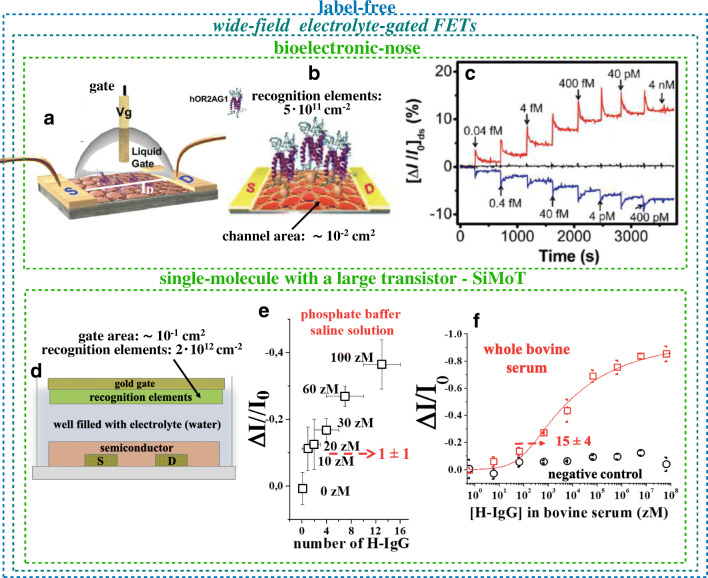
Wide-field electrolyte-gated FETs. **a** Electrolyte-gated FET bioelectronic nose sensor based on plasma-treated bilayer graphene whose outmost surface is conjugated with human olfactory receptors 2AG1 (hOR2AG1). **b** Picture detailing the biofunctionalized graphene FET channel (region between source and drain contacts) whose area is ca. 10^−2^ cm^2^. The density of hOR2AG1 receptors is ca. 5 × 10^11^ cm^−2^. **c** Responses as the fractional change of* I*_D_, Δ*I*/*I*_0_, of the bioelectronic nose upon exposure to different concentrations of the amyl butylate odorant. The red line is measured on an oxygen-plasma-treated graphene surface while the blue line is relevant to an ammonia-plasma-treated surface. The black trace is the response of pristine graphene. (Panels** a**,** b**, and** c** are reprinted with permission from Ref. [33] Copyright 2012 American Chemical Society). **d** Schematic of the single molecule with a large transistor (SiMoT) device structure; the gold gate area is ca. 5 × 10^−1^ cm^2^ while the density of anti-human immunoglobulin G is ca. 2 × 10^12^ cm^−2^. **e** Human immunoglobulin G (H-IgG) curve in phosphate-buffered saline solution in the 0–100 zM range is shown as the fractional change of* I*_D_, Δ*I*/*I*_0_. The x-axis bars are the Poisson errors while y-axis ones are the reproducibility errors. **f** Dose curve of human immunoglobulin G added into whole real bovine blood serum (red squares) in the 0.6 zM to 6 × 10^7^ zM range. In the control experiment human immunoglobulin M was used (black circles) in place of the specific recognition element anti-human immunoglobulin G used for the sensing. The continuous red line is the result of the modelling based on Poisson distribution probability, which is suitable to account for single binding events. All the data points are the average over three replicates measured on different gates and different transistors. Error bars are taken as one standard deviation. (Panels** d **and** f** are reprinted with permission from Ref. [35] Copyright 2018 Springer Nature)

An EG-FET comprises a channel (S and D contacts covered by a SC) whose conductivity is controlled by the gate electrode (G) through a dielectric electrolyte that can even be deionized water [40]. Figure 4a shows a graphene EG-FET bioelectronic sensor of odors. The outermost layer of a graphene double-layer channel material is functionalized with 2AG1 human olfactory receptors (hOR2AG1), capable of selectively binding to amyl butyrate, an odorant marker (Fig. 4b). A p-type layer (n-type) is obtained by exposing the graphene surface to an oxygen plasma (ammonia plasma). Both are used to chemically bind the recognition elements. The ca. 5 × 10^9^ olfactory receptors correspond to a density of 5 × 10^11^ cm^−2^ and result in an equivalent concentration of recognition elements of ca. 50 pM in 100 μl. The gating electrolyte is a pH 7.4 phosphate-buffered saline solution. Figure 4c shows relative variations of the drain current responses as a function of the exposure to different concentrations (40 aM–400 pM) of amyl butylate. Interestingly, the p-type (red trace) and the n-type (blue trace) graphene responses show opposite signs, while the pristine non-binding graphene (black trace) is taken as the reference noise level. A limit of detection as low as 40 aM and a rapid response time of less than 1 s are recorded. The label-free sensing mechanism is ascribed to a structural rearrangement of the olfactory receptor that becomes negatively charged upon selectively binding the analyte. This electrostatic change involving only one step produces a positive charge accumulation in the graphene channel and an increase (decrease) of the drain current* I*_D_ in the p-type (n-type) graphene channel.

In the single molecule with a large transistor (SiMoT) electrolyte-gated organic FET (EGOFET), the gate is biofunctionalized [41] with 2 × 10^12^ recognition elements covalently attached to a 0.5 cm^2^ gold gate; this is equivalent to ca. 10 nM recognition elements available for the binding that takes place on the minute timescale in 100 μl. Figure 4d shows the basic structure of the device while Fig. 4e shows the human immunoglobulin G (H-IgG) protein assay in the 0–100 zM concentration range. Anti-H-IgG-specific capture antibodies served as recognition elements. The responses measured at 10 zM and 20 zM (1 ± 1 particles) are beyond the limit of detection and prove that a wide-field EGOFET can detect a single protein. The SiMoT EGOFETs can also perform label-free detections at the physical limit in a real biofluids, too (Fig. 4f). Bovine blood serum combined with different aliquots of H-IgG analyte protein is assayed (red squares) while human immunoglobulin  M served as non-recognition elements in the control experiment (back circles). A limit of detection of 250 zM, corresponding to 15 ± 4 proteins, was obtained. The SiMoT EGOFET could also detect at the physical limit immunoglobulin M, C-reactive protein in saliva, and HIV p24 [38, 42]; unpublished data are available on the detection of MUC1 and a DNA probe [39].

While investigations into collective effects enabling the signal amplification needed to enable EGOFET bioelectronic sensors to detect 1–10^3^ binding events with a wide surface comprising billions to trillions of recognition elements are in progress, a reasonable explanation involving a combination of capacitive coupled FET transduction and hydrogen-bonding network in self-assembled monolayers has been proposed. In fact, when EGOFETs are operated as capacitive coupled voltage amplifiers, they can amplify the signal produced by the immunometric detection of antigens by a factor 10^3^ times larger than homologous potentiometric electrochemical sensors [26, 27]. Also, a hydrogen-bonding network triggers electrostatic collective interactions that propagate the initial defect generated by the single affinity binding. Consequently, the work function changes over a broad area, thus generating an amplification of the sensing signal [26, 35].

While this technology is just at the level of a proof of principle, the reproducibility error, computed as one standard deviation, was less than 4% [35]. The main drawback of this technology concerns the fact that this new approach is at a very early transfer readiness level.

## Outlook

The single-molecule technologies reviewed span from far-field fluorescent microscopy, near-field plasmonic and electronic approaches as well as wide-field capturing assays that include arrays of microreactors or microwells, through to wide-field electrolyte-gated FETs. While the first technologies were introduced to set the stage, the wide-field capturing and the wide-field approaches are addressed as new trends. Their figures of merit evidence that far- and near-field technologies, while capable of inspecting single-molecule interactions, are generally not suitable for clinical assays as they cannot detect at attomolar or zeptomolar concentrations. The already commercialized wide-field capturing approaches are very well suited for clinical analysis and are opening the path to the widespread use of personalized and precision medicine in everyday clinical practice. The more recently emerged EG-FETs, while still being lab-based single device prototypes, uniquely combine the ability to sense at the single-molecule level in a bulk real biofluid with no need for a labelling step. Potentially, they can be developed into an array and, last but not least, they can be produced by scalable large-area low-cost approaches. The sensing mechanism entails capacitive coupled FET transduction modulated by the biolayer where the sensing occurs and a hydrogen bond cooperative effect that amplifies the effect of the single binding event. A new trend toward an all-electronic array-based bioassay that is capable of detecting at the single-molecule level has started but there is still a rather long way to go to prove its viability for clinical assay use. Indeed, while this new technology has very high potential, it is now barely beyond transfer readiness level 3 (TRL 3, proof of concept demonstrated). It is difficult to predict at this stage if it will be able to become a well-assessed technology. An effort in this direction has been financed by the European Commission as an H2020 research and innovation action titled “The single molecule bio-electronic smart system array for clinical testing” (SiMBiT) (https://simbit-h2020.eu). The SiMBiT project aims at producing a bioelectronic smart system leveraging on an existing lab-based proof of concept that can perform single-molecule detection of both proteins and DNA biomarkers. A main goal of the project is the demonstration of TRL 5, namely, if an EG-FET sensor prototype will be able to be validated in a real environment.
